# The prevalence of estrogen receptor-negative breast cancer in Ethiopia

**DOI:** 10.1186/1471-2407-14-895

**Published:** 2014-11-29

**Authors:** Eva Johanna Kantelhardt, Assefa Mathewos, Abreha Aynalem, Tigeneh Wondemagegnehu, Ahmedin Jemal, Martina Vetter, Erdme Knauf, Anne Reeler, Solomon Bogale, Christoph Thomssen, Andreas Stang, Tufa Gemechu, Pietro Trocchi, Bekuretsion Yonas

**Affiliations:** Department of Gynecology and Institute of Clinical Epidemiology, Martin-Luther-University, Ernst-Grube Str. 40, 06097 Halle an der Saale, Germany; Radiotherapy Center, Addis-Ababa-University, Addis Ababa, Ethiopia; American Cancer Society, Atlanta, USA; Department of Gynecology, Martin-Luther-University, Halle an der Saale, Germany; Axios International, Paris, France; Institute of Clinical Epidemiology, Martin-Luther-University, Halle an der Saale, Germany; Department of Pathology, Addis-Ababa-University, Addis Ababa, Ethiopia; Department of Epidemiology, Boston University, School of Public Health, Boston, MA USA

**Keywords:** Breast neoplasms, Africa, Ethiopia, Prognostic factors

## Abstract

**Background:**

In contrast with breast cancers (BCs) in other parts of the world, most previous studies reported that the majority of BCs in sub-Saharan Africa are estrogen-receptor (ER) negative. However, a recent study using the US SEER database showed that the proportion of ER-negative BC is comparable between US-born blacks and West-African born blacks but substantially lower in East African-born blacks, with over 74% of patients Ethiopians or Eritreans. In this paper, we provide the first report on the proportion of ER-negative BC in Ethiopia, and the relation to progesterone-receptor (PgR) status.

**Methods:**

We analysed 352 female patients with ER results available out of 1208 consecutive female BC patients treated at Addis Ababa-University Hospital, Ethiopia, from June 2005 through December 2010. The influences of age, stage, and histology on the probability of ER-negative tumours were assessed by a log-linear regression model.

**Results:**

Of the 352 patients, only 35% were ER-negative. The proportion of ER-negative tumours decreased with advancing age at diagnosis and was not affected by histology or stage. For age, the proportion decreased by 6% for each additional 5 years (stage-adjusted prevalence ratio PR = 0.94, 95% CI: 0.89–1.00). About 31% were ER- and PgR-negative, and 69% were ER- and/or PgR-positive.

**Conclusions:**

Contrary to most previous reports in other parts of sub-Saharan Africa, the majority of patients in Ethiopia are ER-positive rather than ER-negative. These findings are in line with low proportions of ER-negative BCs from East African immigrants within the SEER database, and they have clinical implications for management of BC patients in Ethiopia and other parts of sub-Saharan Africa where ER-status is not ascertained as part of routine management of the disease. Since the majority of patients showed ER-positive BC, Tamoxifen-therapy should be given to all patients even with unknown ER status.

## Background

Knowledge of the estrogen-receptor (ER) status of breast cancer (BC) is essential in making the decision to treat women with Tamoxifen. Population-based estimates of the distribution of the receptor status of BCs can aid treatment decisions, even among women in whom the individual ER status has not been assessed. Often breast tumours in the African setting are described as being aggressive with negative ER status. Results from East Africa showed 76% of the patients in Kenya and more than two thirds of the patients in Tanzania and Uganda were ER-negative [1–3]. The majority of studies from West Africa showed more than half of the patients were ER-negative: in Nigeria and Senegal, 76% [4]; in Ghana, 76% [5], 75% [6] and 53% [7] and in Mali, 61% [8]. One study from Nigeria, one from Uganda, one from Ghana and one large study from South Africa showed lower proportions of ER-negative tumors (35%, 40%, 24% and 37%, respectively) [9–12] (Table 1).

**Table 1:** **Selection of published results of breast cancer hormone receptor-results (from PubMed terms “AFRICA” and “BREAST CANCER” and “HORMONE RECEPTOR” or “ESTROGEN RECEPTOR” searched June 5, 2014)**

	Total (n)	Year of specimen collection	% of negative estrogen receptor status
**East Africa**			
Kenia [1]	120	2001–2007	76
Tanzania [2]	60	1995–1997	67
Uganda [3]	65	1993–2002	65
**West Africa**			
Nigeria/Senegal [4]	507	2004–2005	76
Ghana [5]	75	2007–2008	76
Ghana [6]	100	2006–2011	75
Ghana [10]	51	2007-2010	76
Mali [8]	114	2008–2011	61
Uganda [11]	45	2000-2004	60
Ghana [7]	68	2004–2009	53
Nigeria [9]	133	1996–2007	35
**Southern Africa**			
South African black population [12]	957	2006–2012	37

The discrepancies in the proportion of ER-negative tumours in Africa are thought to reflect the selection bias of cases and methodological problems associated with laboratory procedures, including degradation of tissue during storage. Also low sensitivity of ER-testing and false negative cases can contribute. Possible regional differences in populations may account for the heterogeneous results involving these tumour biological characteristics but this is questioned by studies showing large differences in the ER-positive proportion within the same population [10]. A recent publication by Jemal *et al.* showed that U.S. immigrants from West Africa with BC had high percentages of ER-negative disease (39.5%). U.S. immigrants with BC from East Africa, mainly Ethiopia, had ER-negative BC in only 25.3% similar to U.S.-born whites with BC [13]. To investigate ER-negative proportions of BC patients, we evaluated for the first time patient and tumour characteristics of a case series of female patients diagnosed with invasive BC from Addis Ababa, Ethiopia.

## Methods

Ethical approval for this study was obtained from the Institutional Review Boards of Addis Ababa University Medical Faculty. The study was conducted without individual informed consent because it relied on retrospective data collected as part of routine patient care. The study was a hospital-based cohort study at the radiotherapy centre of Addis Ababa University Hospital. Women with a histologically verified primary diagnosis of invasive BC between 1 June 2005 and 31 May 2010 who consulted the radiotherapy department at Tikur Anbessa Hospital Addis Ababa were included. The files of BC patients were retrieved manually. All demographic, clinical and pathological characteristics were documented from patients’ files containing physicians’ notes, pathology reports and referral letters.

According to international coding standards for cancer registries [14], the date of incidence was defined as the first consultation at a hospital for the cancer in question or later diagnostic confirmation (e.g. diagnosis by a physician, pathology, date of death). Information about tumour size (T) and nodal status (N) was used to derive the AICC/UICC stage [15]. All staging information mentioned within the first three months after primary pathological information was used for TNM staging [16]. In the case of a clinical T4 description, this was given priority over any pathological Tx. In case of neoadjuvant chemotherapy before the operation, clinical T assessed before chemotherapy was used for the T-stage. TNM classification recommends the removal of at least six lymph nodes (LN) of the axilla. This number of 6 LN was not always described in the pathology report. Therefore, we decided to classify N1 if >50% of the removed nodes were positive. In cases of 4–9 or 10+ positive nodes, we coded N2 or N3. Otherwise, Nx was documented (in line with the European Network of Cancer Registries recommendations [17]). M-stage: The presence of radiologically confirmed distant metastasis at diagnosis was considered to be M1.

In general, diagnosis of BC was obtained by fine-needle aspiration cytology (FNAC) or by surgical biopsy. Early cases were usually operated and therefore tumour material for histology was available. More advanced, inoperable cases or recurrences were usually diagnosed by FNAC only. Therefore, some patients had tumor material for histology, and some only had cytologic material by FNAC available for ER determination and grading. Estrogen receptor staining was introduced in a prospective standardized manner within the independent Ethiopian BC project separately funded and initiated by AstraZenaca Ltd., England, facilitated by the Axios Foundation, France, 2005–2010. Immunohistochemistry was done soon after the specimen were obtained using the estrogen antibody ER 6 F11 and progesterone antibody PGR 312 with the Menarini detection kit according to the standard protocol (A. Menarini diagnostics International®, Firenze, Italy). Evaluation of the staining was done by experienced pathologists in the department according to guidelines assessing any positive stained cell (>1%) as positive ER status [18].

We estimated the percentage (hereafter referred to as “prevalence”) of ER status within the total of ER-stained tumours and among subgroups of clinical importance. To assess the influence of age on the prevalence of ER-negative findings, we estimated prevalence ratios (PR) and corresponding 95% confidence intervals by using a log-linear regression model. Analyses were done using SAS® statistical software (SAS Inc., Cary, NC, USA), Version 9.3.

## Results

The largest proportion of the study population of 1208 female patients was between 30–39 years old (35%). The proportion of patients from Addis Ababa (46%) was slightly higher than the proportion from non-Addis Ababa; the largest proportion of patients presented at stage 3 (36%), mostly without distant metastases at diagnosis (82%). The majority had ductal histology (78%). We compared these characteristics between the total population of 1208 patients and the subgroup of 352 patients with available ER results. The distribution of place of origin, menopausal status, histology and adjuvant therapy was similar. Patients with ER results available were more often under 30 or over 60 years, and also tended to have a higher stage at diagnosis than patients with ER results not available. Due to the preferred procedure of staining FNAC to determine ER, patients with ER results available had more often FNAC done for their pathological diagnosis (Table 2).

**Table 2:** **Clinical and pathological characteristics of the study population**

Parameter	ER available: N (column%)	ER not available: N (column%)
Total population n = 1213	352 (100.0)	861 (100.0)
**Place of origin**		
Addis Ababa	183 (52.0)	405 (47.0)
Non-Addis Ababa	140 (39.8)	375 (43.6)
unknown	29 (8.2)	81 (9.4)
**Age (years)**		
<30	56 (15.9)	106 (12.3)
30–39	114 (32.4)	331 (38.4)
40–49	83 (23.6)	231 (26.8)
50–59	53 (15.1)	139 (16.1)
≥60	46 (13.1)	54 (6.3)
**Menopausal status**		
Premenopausal	145 (41.2)	345 (40.1)
Postmenopausal	155 (44.0)	374 (43.4)
Unknown status	52 (14.8)	142 (16.5)
**Stage (UICC)**		
1 and 2	35 (9.0)	153 (17.8)
3	168 (47.7)	288 (33.4)
4	71 (20.2)	72 (8.4)
Unknown	78 (22.2)	348 (40.4)
**Distant Metastasis**		
No (M0)	236 (67.0)	666 (77.4)
Yes (M1)	116 (33.0)	195 (22.6)
**Histology**		
Ductal	265 (75.3)	681 (79.1)
Lobular	20 (5.7)	39 (4.5)
other/unspecified	67 (19.0)	141 (16.4)
**Patho-specimen for diagnosis**		
FNAC^+^	127 (36.1)	27 (3.1)
Tumour specimen	193 (54.8)	659 (77.0)
unknown	32 (9.1)	174 (20.2)
**Basis of hormone receptor diagnosis**		
Primary operation	174 (49.4)	Not applicable
Recurrence	106 (30.1)	
unknown	72 (20.5)	

^+^FNAC – fine needle aspiration cytology.

Within the subgroup of patients with ER results available, 34.7% (95% CI 28.9–38.8%) had ER-negative findings. The prevalence of ER-negative findings was associated with patients age. Based on a log-linear regression model, the estimated stage-adjusted prevalence of ER-negative findings decreased for each additional 5 years of age by 6% (PR = 0.94, 95% CI: 0.89–1.00), note the CI was close to 1.

Progesterone receptor was positive in 72% (162/224) of ER-positive tumours and 12% (14/120) of ER-negative tumours. None of the other patient characteristics, including place of origin that was not Addis Ababa, stage and histology, was associated with the prevalence of ER-negative results (Table 3, adjusted risk ratios). Results from the FNAC specimen as compared to the tumour specimen had a lower age- and stage-adjusted prevalence-ratio for ER negativity (PR = 0.71; 95% CI: 0.51–0.98).

**Table 3:** **ER results among different groups of patients**

Parameter	ER: N (%) 352 stained tumours			
	Positive (% in row or mean and standard deviation)	Negative (% in row or mean and standard deviation)	ER-negative vs. ER-positive crude RR (95% CI)	ER-negative vs. ER-positive adjusted* RR (95% CI)
Total population n = 352	230 (65.3)	122 (34.7)		
Age [years] (mean and standard deviation)	43.0 (13.7)	40.1 (12.6)	Increase 5 yrs 1.02 (0.98–1.01)	Increase 5 yrs 0.94 (0.89–1.00)
Tumour size [cm] (mean and standard deviation; n = 168 results available)	5.47 (3.3)	6.06 (3.3)	Increase 1 cm 1.03 (0.98–1.08)	Increase 1 cm 1.03 (0.97–1.08)
**Place of origin**				
Addis Ababa	123 (67.2)	60 (32.8)	Reference	Reference
Non-Addis Ababa	89 (63.6)	51 (36.4)	1.11 (0.82–1.50)	1.03 (0.76–1.41)
Unknown	18 (62.1)	11 (37.9)	1.16 (0.69–1.93)	1.10 (0.66–1.82)
**Menopausal status**				
Premenopausal	89 (61.4)	56 (38.6)	1.17 (0.87–1.59)	0.81 (0.54–1.23)
Postmenopausal	104 (67.1)	51 (32.9)	Reference	Reference
unknown	37 (71.2)	15 (28.8)	0.88 (0.54–1.42)	0.62 (0.36–1.07)
**Stage (UICC)**				
1 and 2	24 (68.6)	11 (31.4)	0.89 (0.53–1.52)	0.92 (0.54–1.56)
3	109 (64.9)	59 (35.1)	Reference	Reference
4	48 (67.6)	23 (32.4)	0.92 (0.62–1.37)	0.96 (0.65–1.42)
Unknown	49 (62.8)	29 (37.2)	1.06 (0.74–1.51)	1.06 (0.75–1.51)
**Histology**				
Ductal	171 (64.5)	94 (35.5)	Reference	Reference
Lobular	12 (60.0)	8 (40.0)	1.13 (0.64–1.98)	1.05 (0.60–1.85)
Other/unspecified	47 (70.2)	20 (29.8)	0.84 (0.56–1.26)	0.83 (0.56–1.24)
**Progesterone receptor**				
Positive	162 (92.0)	14 (8.0)	Reference	Reference
Negative	62 (36.9)	106 (63.1)	7.93 (4.74–13.28)	7.81 (4.66–13.09)
Unknown	6 (75.0)	2 (25.0)	3.14 (0.86–11.55)	3.47 (0.94–12.78)
**Basis of hormone receptor diagnosis**				
Primary operation	117 (67.2)	57 (32.8)	Reference	Reference
Recurrence	62 (58.5)	44 (41.5)	1.12 (0.96–1.31)	1.08 (0.91–1.27)
Unknown basis	51 (70.8)	21 (29.2)	0.89 (0.59–1.35)	0.87 (0.58–1.33)
**Patho-specimen for diagnosis**				
FNAC^+^	92 (72.4)	35 (27.6)	0.67 (0.48–0.94)	0.71 (0.51–0.98)
Tumour specimen	114 (59.1)	79 (40.9)	Reference	Reference
Unknown	24 (75.0)	8 (25.0)	0.61 (0.33–1.14)	0.61 (0.33–1.16)

^+^FNAC – fine needle aspiration cytology.

*Risk ratios were adjusted for age and stage, unless this was the variable of interest, RR for age was not adjusted.

## Discussion

In our study, the tumour biology of female Ethiopian BC patients was favourable: only a small proportion, 34.7%, of patients had ER-negative BC (results available, n = 352). Older age predicted lower proportions of ER negativity (decrease in 5 years: 6.4%). All other patient characteristics, especially advanced stage, histology and ER results from local recurrence, did not predict ER negativity. Results from FNAC were less often ER-negative than results from tumour specimens.

Our study is in line with the findings of Jemal *et al.*[13], showing that U.S. immigrants from East Africa have a low proportion of ER-negative breast tumours, and studies from Nigeria, Ghana, Uganda and from South Africa that found <35% of ER negativity in BC patients from Africa [9–12]. The majority of studies on tumour biology in Africa found large proportions of ER-negative tumours. Reasons for these findings might be true differences in the population due to environmental and genetic factors. However, it cannot be ruled out that these findings might also reflect bias due to lack of standardized staining and reading procedures, possible antigen degradation of archived material, and patient selection.

A limitation of our study is we only tested ER status on 29% of all the cases. However this group did not differ much from the total cohort of 1208 cases in clinical and pathological characteristics. Another limitation of our study is using results from FNAC staining among 36.1% of the cases instead of the gold standard histologic staining. In the cohort, FNAC showed a lower proportion of ER-negative results compared to the gold standard of histologic staining. Even though FNAC is not the gold standard, we believe FNAC staining does accurately reflect the immunohistochemical results for the tumour as comparative studies have shown [19–21]. Since FNAC is done more often in advanced cancer cases, the group with available ER results has a higher proportion of advanced stage patients, i.e. stage 4 (20.2% compared to 8.4%). Additionally, ER was sometimes stained on material from local recurrence. Jabbour et al. in their review article describe a 20% variation of ER expression between local recurrence and primary tumour, rather than switching from ER-positive to ER-negative expression in recurrences. Also, more advanced stages rather tend to be receptor-negative more frequently [22]. Therefore, our patient cohort would be expected to have high false ER-negative expression due to some results originating from recurrence material compared to the original primary tumours. Since our findings show a low ER-negative proportion, this proportion would even be lower in the primary and early stage tumours often described in studies from the Western world. Therefore, our results would even overestimate the proportion of ER-negative findings.

## Conclusion

Our study showed that the proportion of ER-negative BC is low in Ethiopia (35%) using 352 BC cases with results available from a large consecutive cohort. These findings are in line with low proportions previously reported from East-Africa-born US-patients and other studies from Africa [10, 11, 13]. In case of low proportions of ER-positive cases in the routine, suspicion should be raised and a reference laboratory consulted. Hormone receptor staining is not often available in Ethiopia due to limited resources. From our findings, we conclude that patients with unknown receptor status are more likely to be ER-positive and therefore should be treated with Tamoxifen. This is especially true for older patients with lower chances of ER-negative disease.

## Abbreviations

BC: Breast cancer
ER: Estrogen receptor
FNAC: Fine-needle aspiration cytology
PgR: Progesterone receptor.

## References

[1] Bird PA, Hill AG, Houssami N (2008). Poor hormone receptor expression in East African breast cancer: evidence of a biologically different disease?. Ann Surg Oncol.

[2] Mbonde MP, Amir H, Akslen LA, Kitinya JN (2001). Expression of oestrogen and progesterone receptors, Ki67, p53 and BCL-2 proetins, cathepsin D, urokinase plasminogen activator and urokinase plasminogen activator-receptors in carcinomas of the female breast in an African Population. East African Medical Journal.

[3] Nalwoga H, Arnes JB, Wabinga H, Akslen LA (2007). Frequency of the basal-like phenotype in African breast cancer. APMIS.

[4] Huo D, Ikpatt F, Khramtsov A, Dangou J, Nanda R, Dignam J, Zhang B, Grushko T, Zhang C, Oluwasola O, Malaka D, Malami S, Odetunde A, Adeoye AO, Iyare F, Falusi A, Perou CM, Olopade OI (2009). Population differences in breast cancer: survey in indigenous African women reveals over-representation of triple-negative breast cancer. J Clin Oncol.

[5] Stark A, Kleer CG, Martin I, Awuah B, Nsiah-Asare A, Takyi V, Braman M, Quayson SE, Zarbo R, Wicha M, Newman L (2010). African ancestry and higher prevalence of triple-negative breast cancer: findings from an international study. Cancer.

[6] Pang J, Toy KA, Griffith KA, Awuah B, Quayson S, Newman LA, Kleer CG (2012). Invasive breast carcinomas in Ghana: high frequency of high grade, basal-like histology and high EZH2 expression. Breast Cancer Res Treat.

[7] Ohene-Yeboah M, Adjei E (2012). Breast cancer in Kumasi, Ghana. Ghana Med J.

[8] Ly M, Antoine M, Dembele AK, Levy P, Rodenas A, Toure BA, Badiaga Y, Dembele BK, Bagayogo DC, Diallo YL, Kone AA, Callard P, Bernaudin J, Diallo DA (2012). High incidence of triple-negative tumors in Sub-Saharan Africa: a prospective study of breast cancer characteristics and risk factors in Malian Women Seen in a Bamako University Hospital. Oncology.

[9] Adebamowo CA, Famooto A, Ogundiran TO, Aniagwu T, Nkwodimmah C, Akang EE (2008). Immunohistochemical and molecular subtypes of Breast Cancer in Nigeria. Br Can Res Tr.

[10] Adjei EK, Owusu-Afriyie O, Awuah B, Stalsberg H (2014). Hormone receptors and Her2 expression in breast cancer in sub-Saharan Africa. A comparative study of biopsies from Ghana and Norway. Breast J.

[11] Roy I, Othieno E (2011). Breast carcinoma in Uganda: microscopic study and receptor profile of 45 cases. Arch Pathol Lab Med.

[12] McCormack VA, Joffe M, van den Berg E, Broeze N, Dos Santos SI, Romieu I, Jacobson JS, Neugut AI, Schuz J, Cubasch H (2013). Breast cancer receptor status and stage at diagnosis in over 1,200 consecutive public hospital patients in Soweto, South Africa: a case series. Breast Cancer Res.

[13] Jemal A, Fedewa SA (2012). Is the prevalence of ER-negative breast cancer in the US higher among Africa-born than US-born black women?. Breast Cancer Res Treat.

[14] Jensen OM, Parkin DM, MacLennan R, Muir CS, Skeet RG (1991). Cancer Registration: Principles and Methods: IARC Scientific Publication No. 95.

[15] Edge SB (2010). AJCC Cancer Staging Manual.

[16] Sobin L, Gospodarowicz M, Wittekind C (2009). TNM Classification of malignant tumors.

[17] European network of cancer registries (ENCR): *Condensed TNM for Coding the Extent of Disease. ENCR RECOMMENDATIONS*. [http://www.encr.eu/images/docs/recommendations/extentofdisease.pdf]

[18] Goldhirsch A, Wood WC, Gelber RD, Coates AS, Thurlimann B, Senn H (2007). Progress and promise: highlights of the international expert consensus on the primary therapy of early breast cancer 2007. Ann Oncol.

[19] Fernando IN, Powles TJ, Dowsett M, Ashley S, McRobert L, Titley J, Ormerod MG, Sacks N, Nicolson MC, Nash A (1995). Determining factors which predict response to primary medical therapy in breast cancer using a single fine needle aspirate with immunocytochemical staining and flow cytometry. Virchows Arch.

[20] Makris A, Allred DC, Powles TJ, Dowsett M, Fernando IN, Trott PA, Ashley SE, Ormerod MG, Titley JC, Osborne CK (1997). Cytological evaluation of biological prognostic markers from primary breast carcinomas. Breast Cancer Res Treat.

[21] Pegolo E, Machin P, Riosa F, Bassini A, Deroma L, Di Loreto C (2012). Hormone receptor and human epidermal growth factor receptor 2 status evaluation on ThinPrep specimens from breast carcinoma: correlation with histologic sections determination. Cancer Cytopathol.

[22] Jabbour MN, Massad CY, Boulos FI (2012). Variability in hormone and growth factor receptor expression in primary versus recurrent, metastatic, and post-neoadjuvant breast carcinoma. Breast Cancer Res Treat.

[CR23] The pre-publication history for this paper can be accessed here:http://www.biomedcentral.com/1471-2407/14/895/prepub

